# Supplementary material for: The claustrum-prelimbic cortex circuit through dynorphin/κ-opioid receptor signaling underlies depression-like behaviors associated with social stress etiology
Source: Nat Commun. 2023 Nov 30;14:7903. doi: 10.1038/s41467-023-43636-x (PMC10689794; doi:10.1038/s41467-023-43636-x)
Supplement: Supplementary file 1 — Supplementary figures [file 41467_2023_43636_MOESM1_ESM.docx]

**­ Supplementary Information**

**The claustrum-prelimbic cortex circuit through dynorphin/κ-opioid receptor signaling underlies depression-like behaviors associated with social stress etiology**

Yu-Jun Wang^1, 2, 3#^, Gui-Ying Zan^1, 2#^, Cenglin Xu^4#^, Xue-Ping Li^1#^, Xuelian Shu^1, 2#^, Song-Yu Yao^5^, Xiao-Shan Xu^6^, Xiaoyun Qiu^4^, Yexiang Chen^7, 8^, Kai Jin^6^, Qi-Xin Zhou^6^, Jia-Yu Ye^4,8^, Yi Wang^4^, Lin Xu^6*^, Zhong Chen^4*^, Jing-Gen Liu^1, 2, 4, 8*^

^#^These authors contributed equally to this work.

^*^Correspondence: lxu@vip.163.com (Lin Xu); chenzhong@zju.edu.cn (Zhong Chen); [jgliu@simm.ac.cn](mailto:jgliu@simm.ac.cn) (Jing-Gen Liu)

**
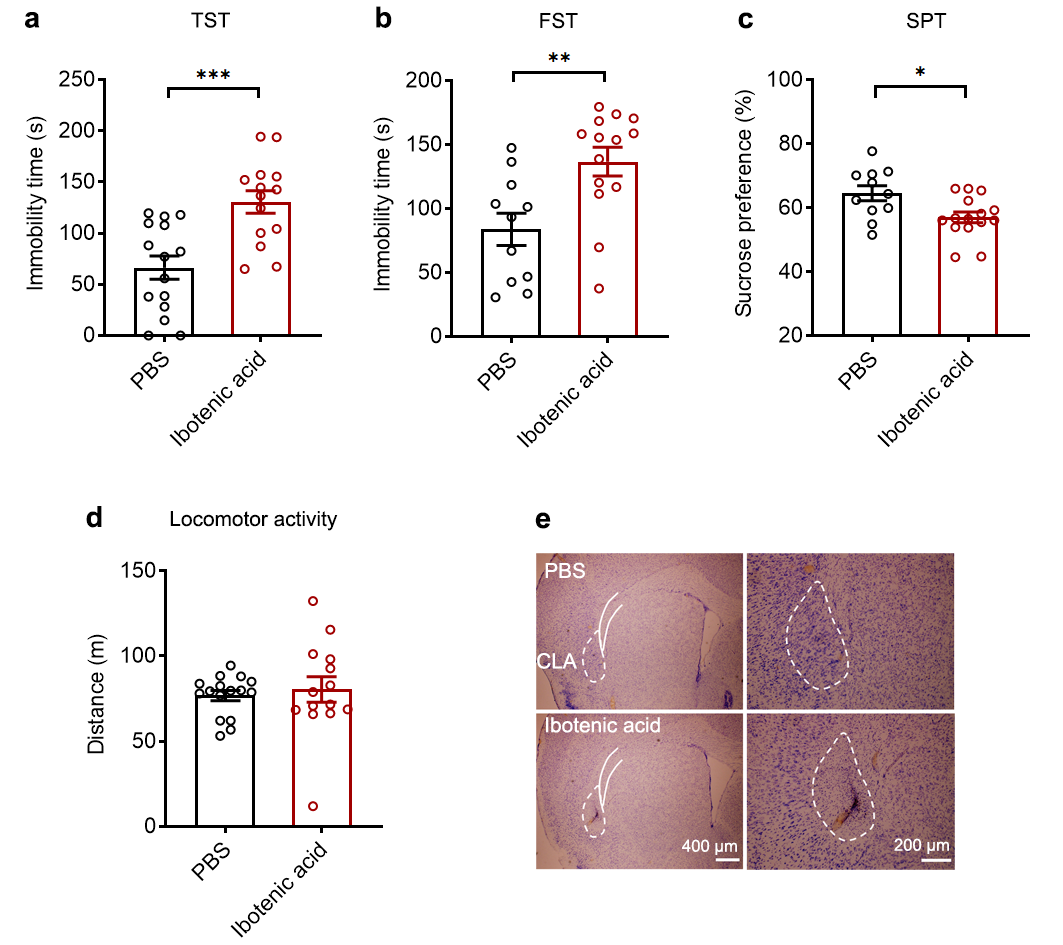
**

**Supplementary Fig. 1 Bilateral lesion of CLA induced depressive-like behaviors.**

**a-d**, Bilateral lesion of the CLA with ibotenic acid significantly increased immobility time in the TST (**a**, *n* = 14-15, *t*_(27)_ = 4.041, *P* = 0.0004. Student’s *t*-test) and FST (**b**, *n* = 11-14, *t*_(23)_ = 3.142, *P* = 0.0046. Student’s *t*-test), and decreased sucrose consumption in the SPT (**c**, *n* = 11-15, *t*_(24)_ = 2.675, *P* = 0.0132. Student’s *t*-test), without affecting mice locomotor activity (**d**, *n* = 14-16, *t*_(28)_ = 0.4625, *P* = 0.6473. Student’s *t*-test). **e**, Nissl staining showed CLA neurons were damaged by ibotenic acid. All data are shown as mean ± s.e.m. ^*^*P* < 0.05, ^**^*P* < 0.01, ^***^*P* < 0.001. Source data are provided as a Source Data file.

**
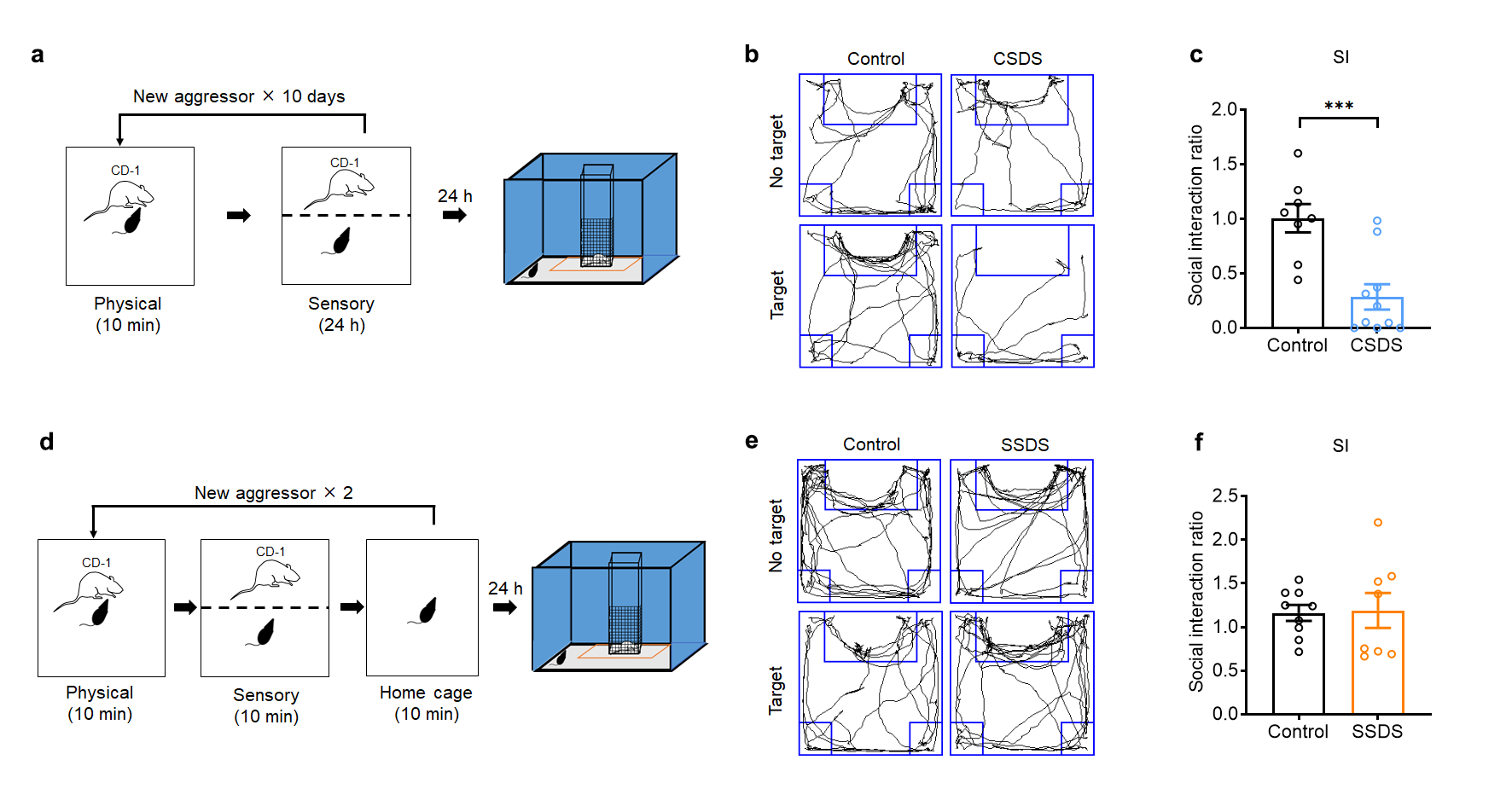
**

**Supplementary Fig. 2 Development of animal models of social defeat stress.**

**a-c**, The CSDS mice model. Schematic diagram of the experimental procedure and behavior test (**a**). Representative activity tracking during social interaction test (**b**) and quantification of the social interaction ratio following CSDS (**c**, *n* = 8-10, *t*_(16)_ = 4.131, *P* = 0.0008. Student’s *t*-test). **d-f**, The SSDS mice model. Schematic diagram of experimental procedure (**d**). Representative activity tracking (**e**) and quantification of the social interaction after SSDS (**f**, *n* = 8-9, *t*_(15)_ = 0.1319, *P* = 0.8968. Student’s *t*-test). All data are shown as mean ± s.e.m. ^***^*P* < 0.001. Source data are provided as a Source Data file.


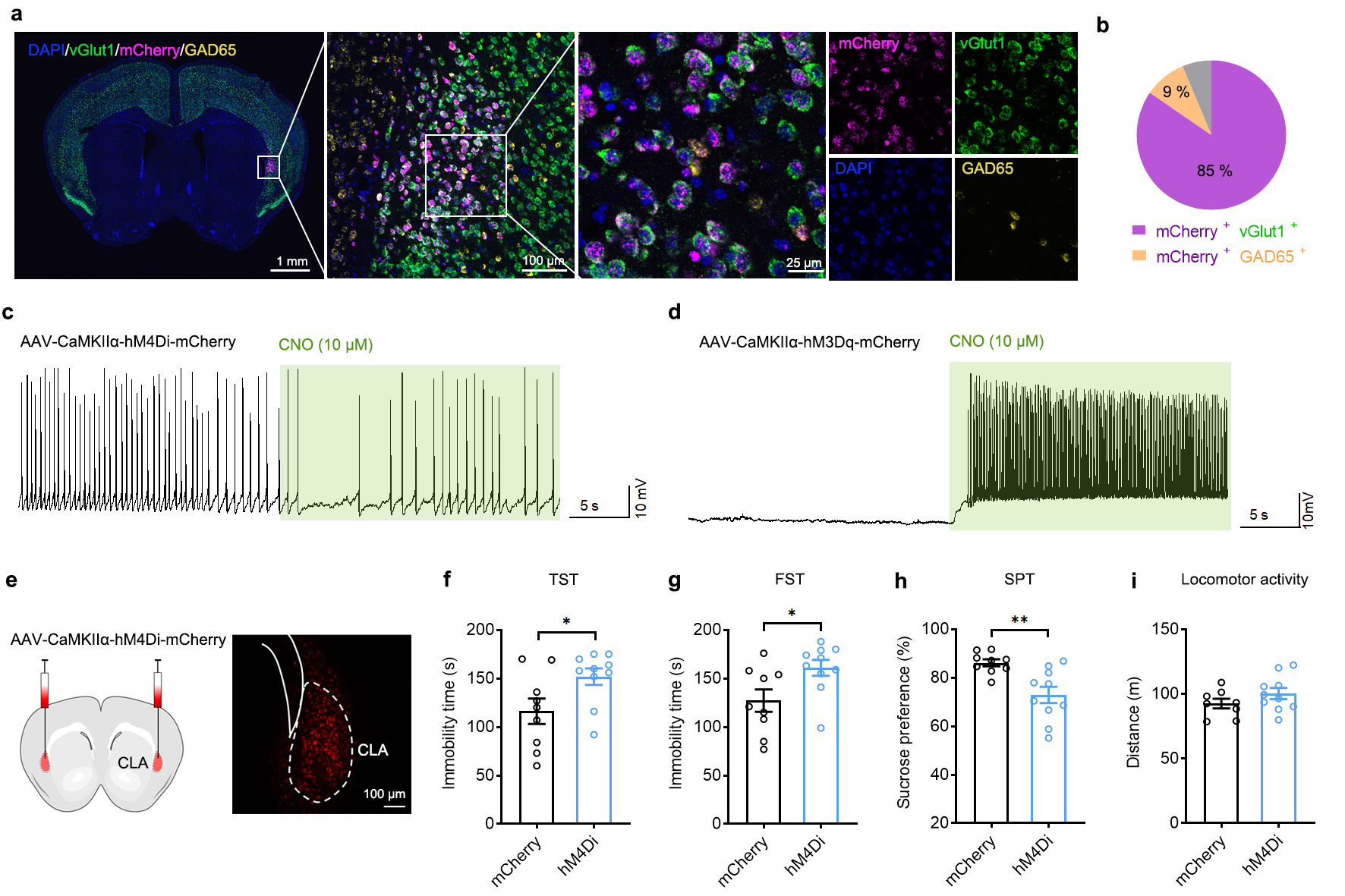
**Supplementary Fig. 3. Chemogenetics enable manipulation of CLA glutamatergic neuronal activity.**

**a, b**, Virus containing a CaMKIIα promoter predominantly expressed in glutamatergic neurons in the CLA. In situ hybridization of mCherry (purple), vGlut1 (green) and GAD65 (yellow) mRNAs reveals that a vast majority of cells expressing AAV-CaMKIIα-hM4Di-mCherry also express vGlut1, but not GAD65 (**a**). Percentages of mCherry^+^ neurons co-labeled with vGlut1 or GAD65 (**b**). Data are shown as mean ± s.e.m from 4 mice, with one to three sections per mouse. **c, d**, The inhibitory and excitatory effects of the hM4Di and hM3Dq on CLA glutamatergic neuronal activity. Representative traces of spontaneous spikes in CLA AAV-CaMKIIα-hM4Di-mCherry (**c**) or AAV-CaMKIIα-hM3Dq-mCherry (**d**) positive neurons with or without focal application of CNO (10 μM) in the ex vivo slices. (**e-i**) Chemogenetic inhibition of the CLA glutamatergic neurons induced depressive-like behaviors in control mice. Left: schematic of CLA injection of AAV-CaMKIIα-hM4Di-mCherry; Right: representative images showing the red fluorescence for the expression of mCherry in CLA (**e**)**.** Chemogenetic inhibition of the CLA glutamatergic neurons increased immobility time in the TST (**f**, *n* = 9-10, *t*_(17)_ = 2.323, *P* = 0.0328. Student’s *t*-test) and FST (**g**, *n* = 9-10, *t*_(17)_ = 2.444, *P* = 0.0257. Student’s *t*-test) and decreased sucrose consumption in the SPT (**h**, *n* = 9-10, *t*_(17)_ = 3.443, *P* = 0.0031. Student’s *t*-test), without affecting mice locomotor activity (**i**, *n* = 8-10, *t*_(16)_ = 1.320, *P* = 0.2055. Student’s *t*-test). All data are shown as mean ± s.e.m. ^*^*P* < 0.05, ^**^*P* < 0.01. Source data are provided as a Source Data file.


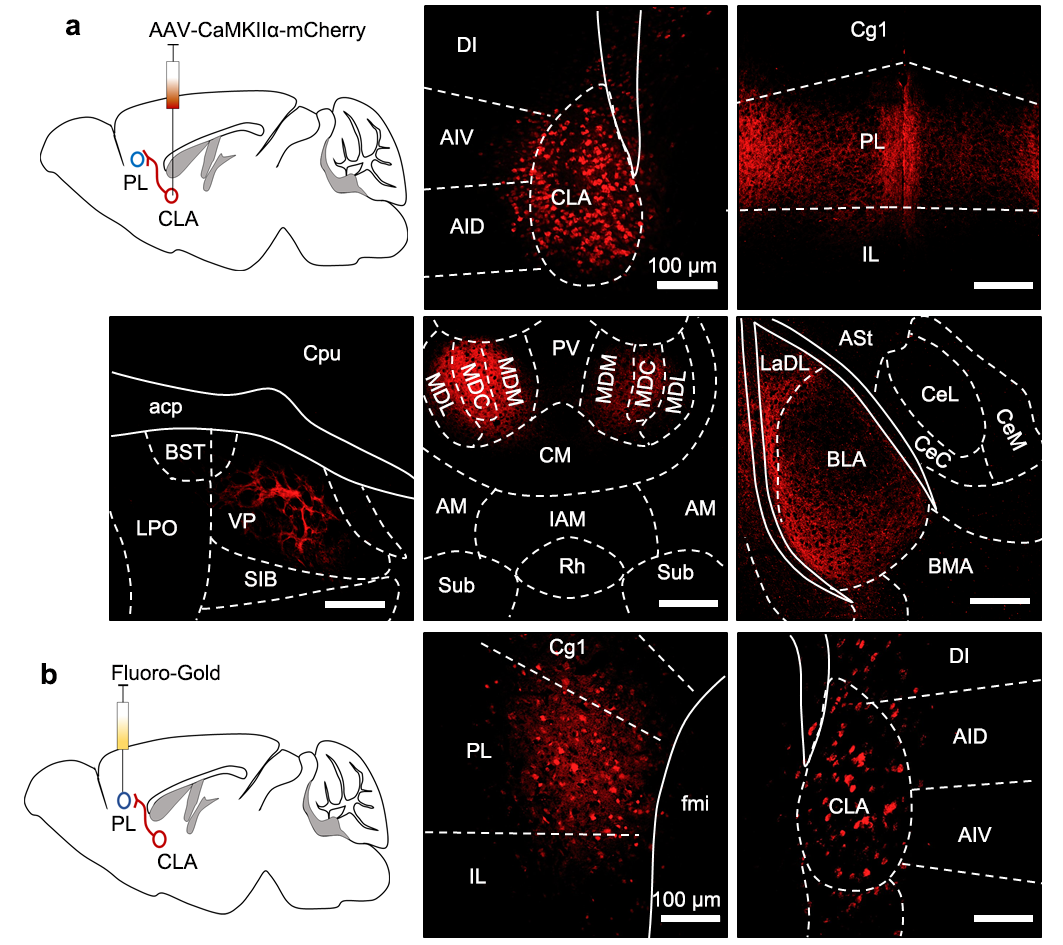


**Supplementary Fig. 4. Tracing of the CLA-PL pathway.**

**a**, Schematic and representative images of CLA injection of AAV-CaMKIIα-mCherry and anterograde viral tracing in the PL, VP, MD and BLA. **b**, Schematic and representative images of PL injection of Fluoro-Gold and retrograde tracing in the CLA.


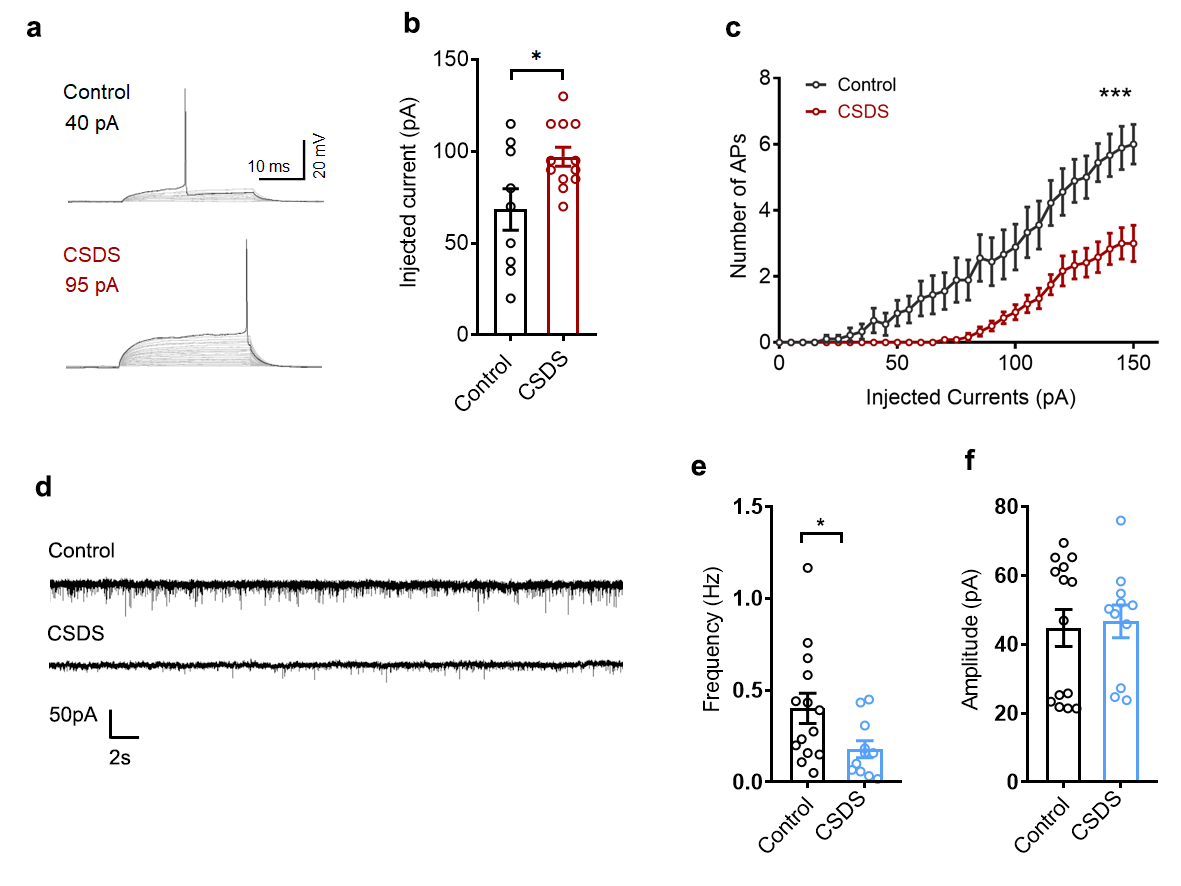


**Supplementary Fig. 5. CSDS decreased the activity of CLA glutamatergic neurons and PL PV interneurons.**

**a, b,** Sample tracers (**a**) and summarized data showing minimal injected current to induce action potential of CLA neurons was increased in CSDS mice (**b**, *n* = 9-12, *t*_(19)_ = 2.520, *P* = 0.0209. Student’s *t*-test). **c,** Quantification of induced action potential numbers at different current pulses from 0 to 150 pA in 5 pA steps (*n*=9-12, *F*_(30, 589)_ = 3.578, *P* < 0.0001. Two-way ANOVA). **d-f,** Sample tracers (**d**) and summarized data showing the frequency of EPSCs recorded in PL PV neurons was decreased in CSDS mice (**e**, *n* = 11-14, *t*(23) = 2.184, *P* = 0.0394. Student’s *t*-test), while the amplitude was not altered (**f**, *n* = 11-14, *t*(23) = 0.2596, *P* = 0.7975. Student’s *t*-test). Source data are provided as a Source Data file.

**
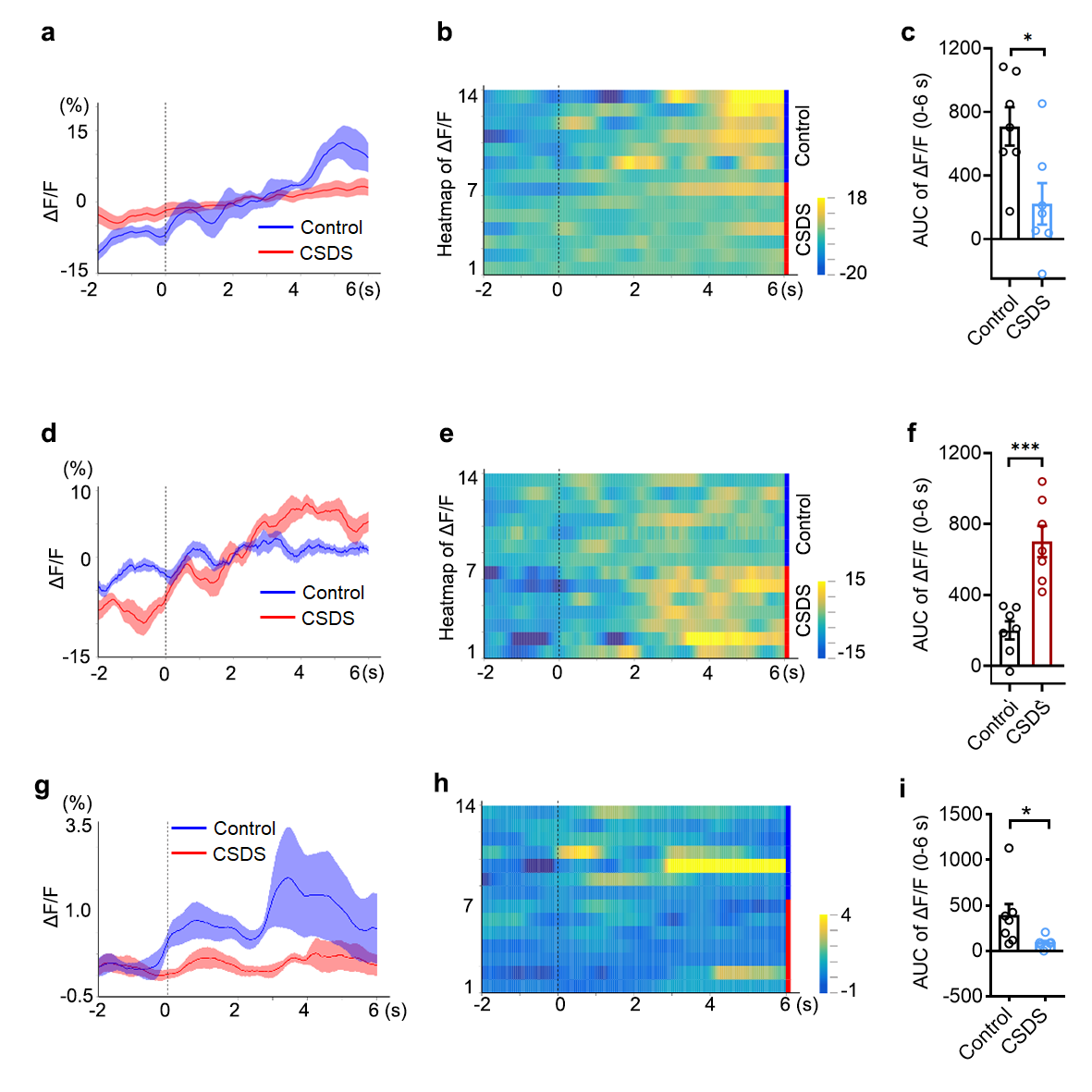
**

**Supplementary Fig. 6. CSDS decreased the real-time activity of CLA glutamatergic neurons and PL PV interneurons, but increased the activity of PL glutamatergic neurons.**

**a, d, g,** Representative traces of Ca^2+^ signals of CLA glutamatergic neurons (**a**), PL glutamatergic neurons (**d**) or PL PV interneurons (**g**) in control mice (blue line) and CSDS mice (red line) during the first social interaction trail (-2s ― +6s). The dashed line showed the onset of the first social interaction. The curves and shaded regions indicate the mean ± s.e.m. n = 7 mice. **b, e, h,** Heatmaps across animals aligned to the first social interaction trail. **c, f, i,** Quantification of area under the curve (AUC, 0 ~ 6 s) of average ΔF/F during the first social interaction trail (**c**, *n* = 7, *t*_(12)_ = 2.737, *P* = 0.018; **f**, *n* = 7, *t*_(12)_ = 4.934, *P* = 0.0003; **i**, *n* =7, *t*_(12 )_ =2.181, *P* =0.0498, Student’s *t*-test). All data are shown as mean ± s.e.m. ^*^*P* < 0.05, ^***^*P* < 0.001. Source data are provided as a Source Data file.


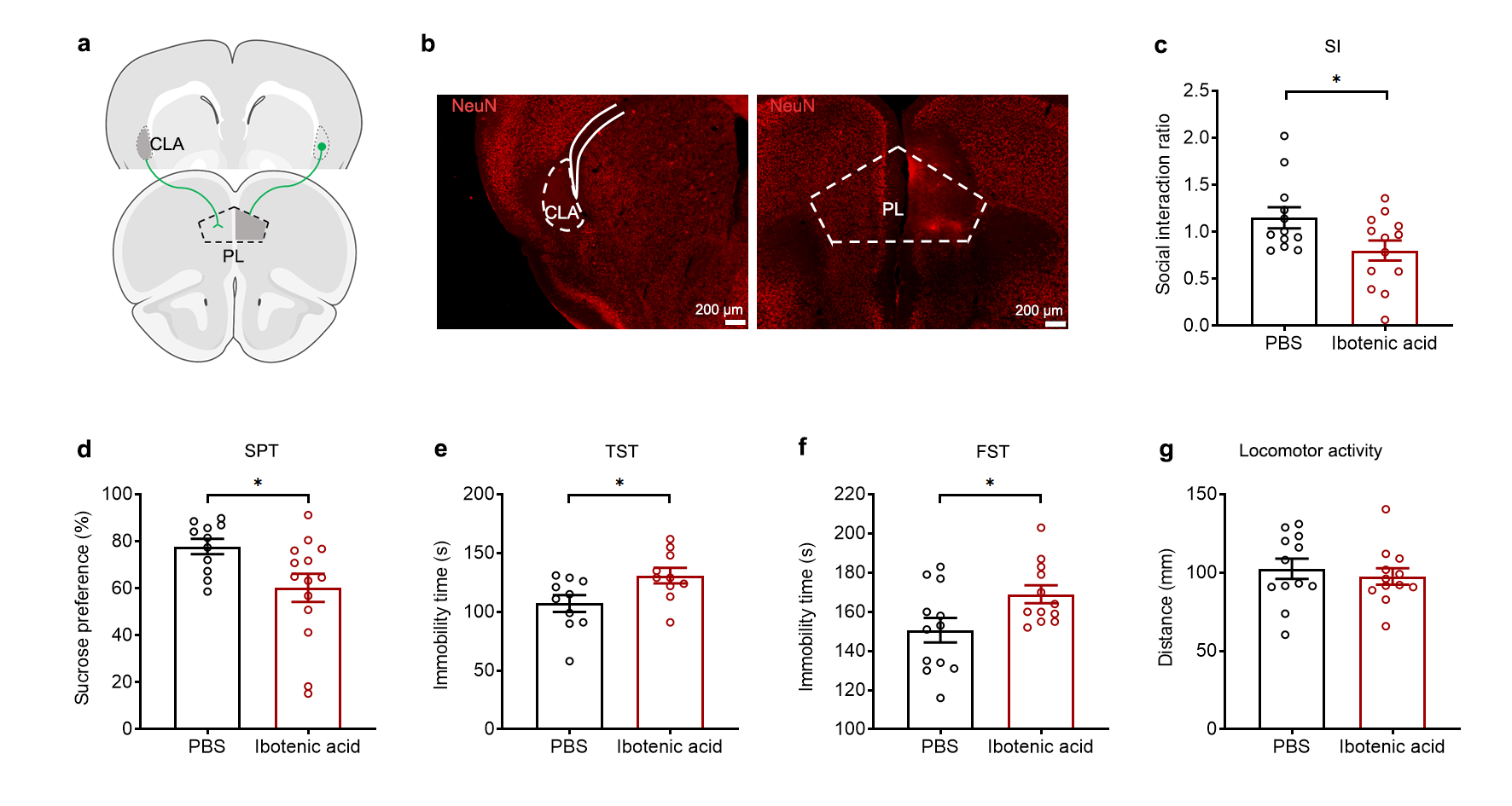


**Supplementary Fig. 7. Disconnection of the CLA-PL projection facilitates depressive-like behaviors induced by SSDS.**

**a**, Schematic illustration of contralateral lesions of CLA and PL. **b**, Representative image of NeuN immunofluorescence staining showing ibotenic acid caused neural lesion in left CLA and right PL. **c-g**, Disconnection of the CLA-PL pathway decreased social interaction (**c**, *n* = 12-13, *t*_(23)_ = 2.253, *P* = 0.0341. Student’s *t*-test), decreased sucrose consumption in the SPT (**d**, *n* = 11-14, *t*_(23)_ = 2.398, *P* = 0.0250. Student’s *t*-test) and increased immobility time in the TST (**e**, *n* = 10, *t*_(18)_ = 2.423, *P* = 0.0262. Student’s *t*-test) and FST (**f**, *n* = 12, *t*_(22)_ = 2.359, *P* = 0.0276. Student’s *t*-test), without affecting locomotor activity (**g**, *n* = 12, *t*_(22)_ = 0.5843, *P* = 0.5649. Student’s *t*-test). All data are shown as mean ± s.e.m. ^*^*P* < 0.05. Source data are provided as a Source Data file.


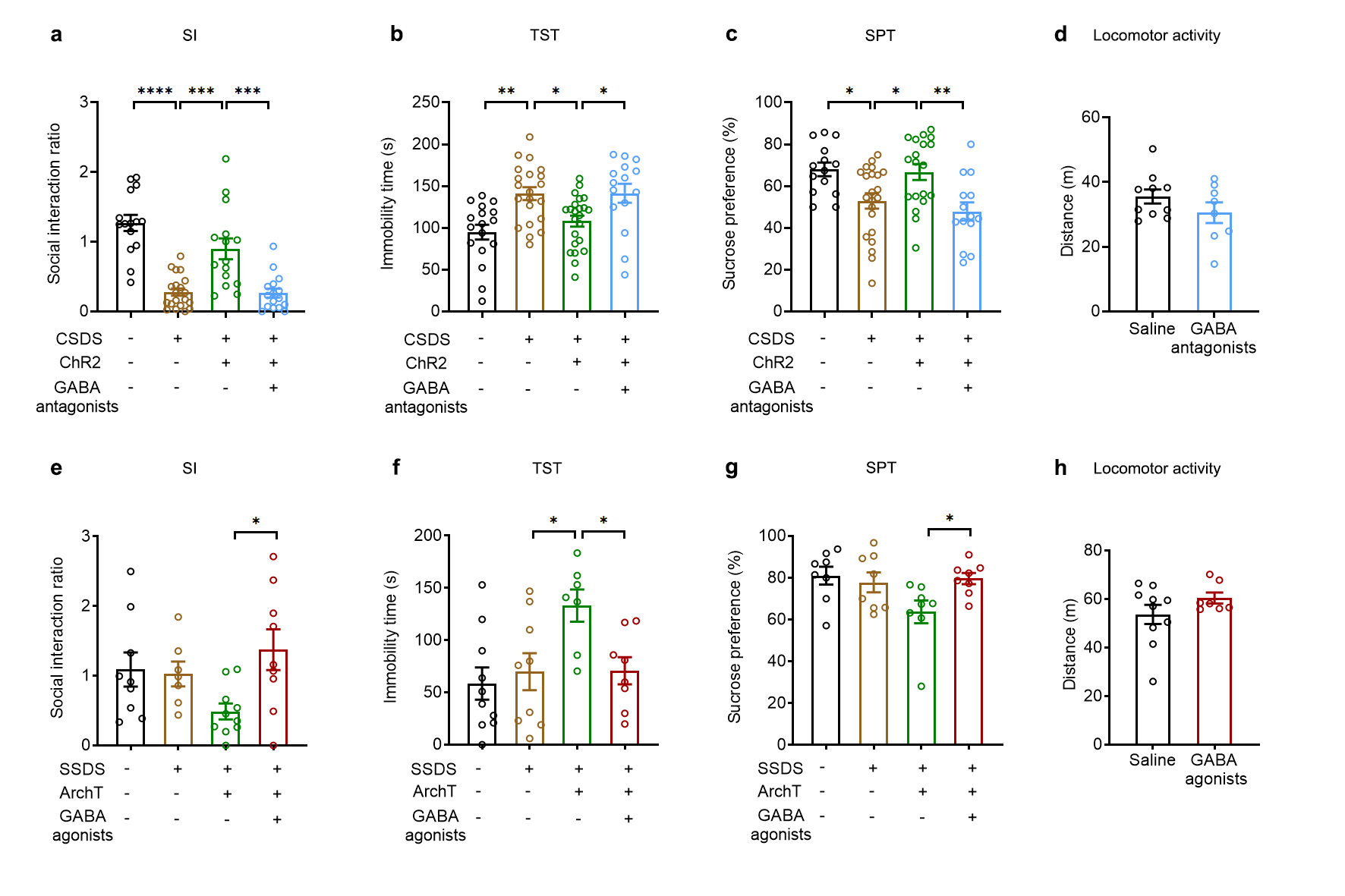


**Supplementary Fig. 8. Manipulation of GABA receptors in the PL modulates depressive-like behaviors.**

**a-d**, Inhibition of the GABA receptors in the PL reversed the anti-depressant effect of photo-activation of the CLA^Glu^-PL pathway. Intra-PL infusion of the GABA antagonists attenuated social interaction ratio (**a**, *n* = 15-20, *F*_(3,62)_ = 25.77, *P* < 0.0001. One-way ANOVA), increased the immobility time in the TST (**b**, *n* = 15-23, *F*_(3,72)_ = 7.589, *P* = 0.0002. One-way ANOVA) and decreased the sucrose consumption in the SPT (**c**, *n* = 14-22, *F*_(3,65)_ = 6.395, *P* = 0.0007. One-way ANOVA), without affecting locomotor activity (**d**, *n* = 8-10, *t*_(16)_ = 1.341, *P* = 0.1988. Student’s *t*-test). **e-h**, Activation of the GABA receptors in the PL blocked the depressive-like behaviors induced by photo-inhibition of the CLA^Glu^-PL pathway. Intra-PL infusion of the GABA agonists increased the social interaction ratio (**e**, *n* = 7-10, *F*_(3,31)_ = 3.133, *P* = 0.0395. One-way ANOVA), decreased the immobility time in the TST (**f**, *n* = 7-10, *F*_(3,30)_ = 4.102, *P* = 0.0149. One-way ANOVA) and increased sucrose consumption in the SPT (**g**, *n* = 8, *F*_(3,28)_ = 3.262, *P* = 0.0361. One-way ANOVA), without affecting mice locomotor activity (**h**, *n* = 7-10, *t*_(15)_ = 1.324, *P* = 0.2052. Student’s *t*-test). All data are shown as mean ± s.e.m. ^*^*P* < 0.05, ^**^*P* < 0.01, ^***^*P* < 0.001, ^****^*P* < 0.0001. Source data are provided as a Source Data file.

**
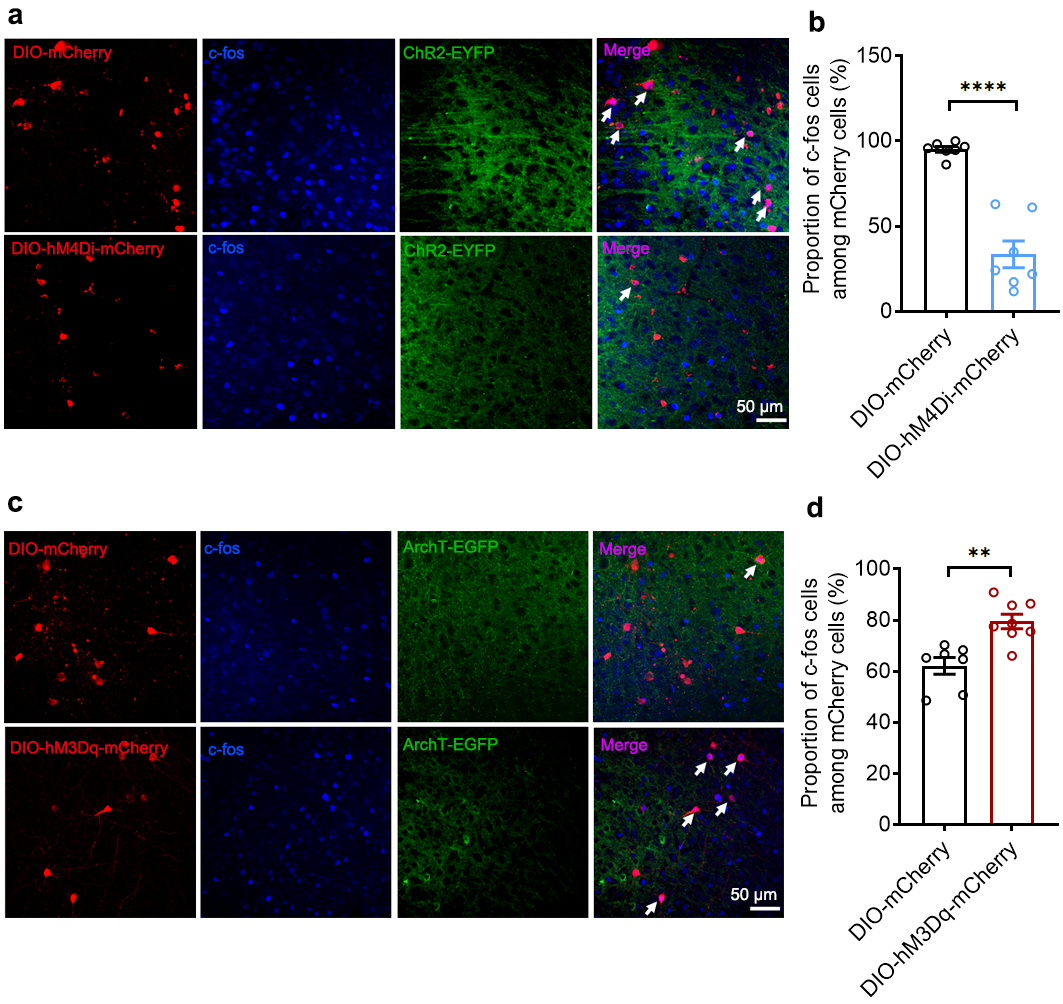
**

**Supplementary Fig. 9. Chemogenetic modulation of PL PV interneurons regulates optogenetic manipulation of CLA-PL projection-induced c-fos expression in the PL.**

**a**, Chemogenetic inhibition of PV interneurons in the PL decreased c-fos expression in DIO-hM4Di-mCherry positive cells while photo-activation of the CLA^Glu^-PL pathway. **b**, Proportion of c-fos positive cells among mCherry positive cells (*n* = 7, *t*_(12)_ = 7.688, *P* < 0.0001. Student’s *t*-test). **c**, Chemogenetic activation of PV interneurons in the PL increased c-fos expression in DIO-hM3Dq-mCherry positive cells while photo-inhibition of the CLA^Glu^-PL projection. **d**, Proportion of c-fos positive cells among mCherry positive cells (*n* = 7-8, *t*_(13)_ = 4.049, *P* = 0.0014. Student’s *t*-test). All data are shown as mean ± s.e.m. ^**^*P* < 0.01, ^****^*P* < 0.0001. Source data are provided as a Source Data file.


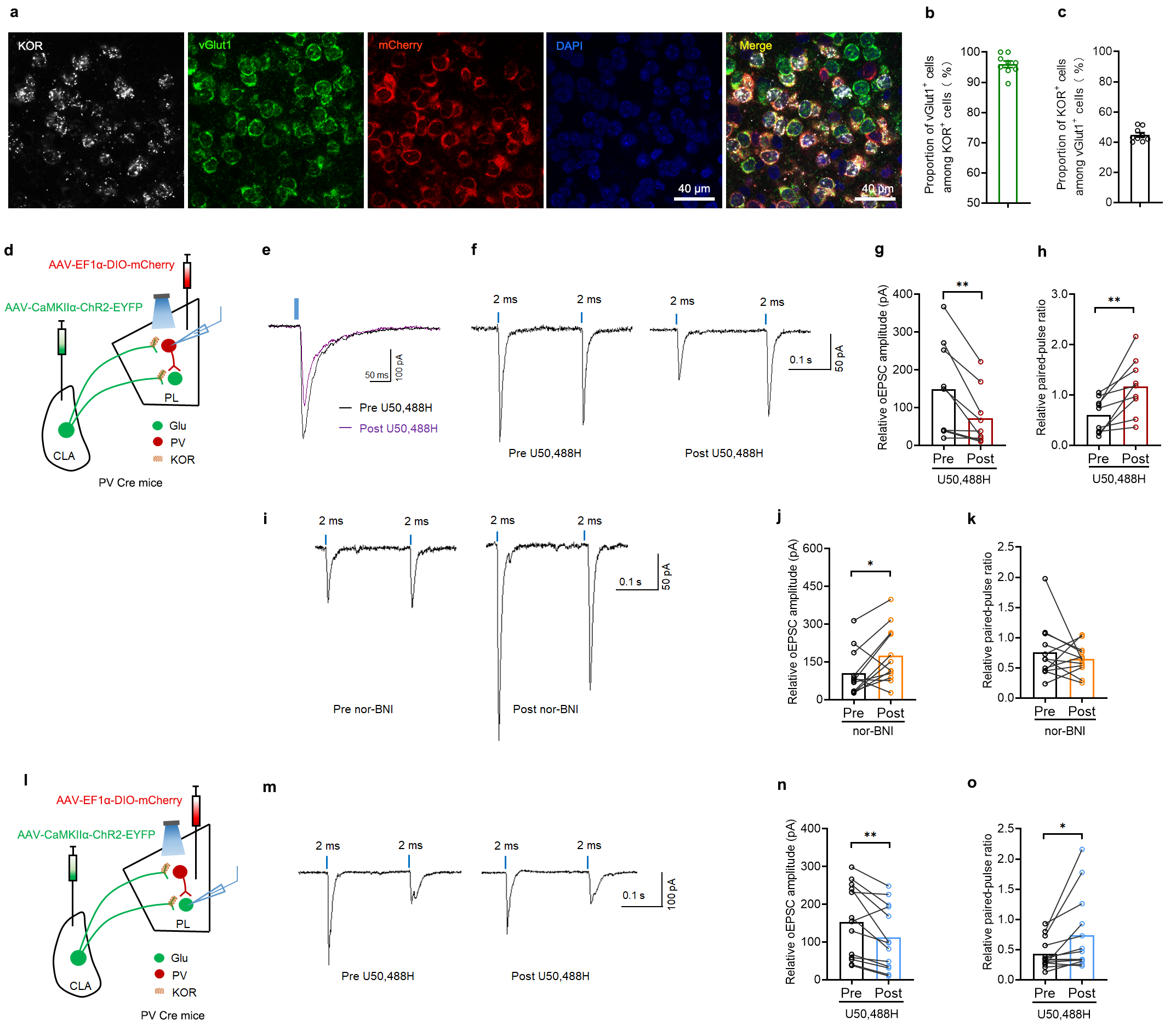


**Supplementary Fig. 10. KORs negatively modulate CLA^Glu^-PL synaptic efficacy.**

**a**, Representative images of KOR mRNA (white), vGlut1 (green), ChR2-mCherry (red) and DAPI (blue) in the CLA after injection of AAV-CaMKIIα-ChR2-mCherry into the CLA. **b**, Proportion of vGlut1 positive cells among KOR positive cells. **c**, Proportion of KOR positive cells among vGlut1 positive cells. Data were obtained from 9 sections from 3 mice. **d**, **l,** Schematic of in vitro whole-cell slice electrophysiology recording to testing the function of KORs in CLA-PL circuit. **e**, **f**, **i,** Representative traces of oEPSC of PL PV interneurons in response to optical stimulation of CLA glutamatergic afferents before and after U50,488H (**e, f**) or nor-BNI (**i**) application. **g**, **h**, U50,488H application decreased oEPSCs amplitude (**g**, *n* = 9, *t*_(8)_ = 3.416, *P* = 0.0091. Paired student’s *t*-test) and increased PPR ratio (**h**, *n* = 9, *t*_(8)_ = 3.759, *P* = 0.0056. Paired student’s *t*-test) in PL PV interneurons. **j**, **k,** nor-BNI application increased oEPSCs amplitude (**j**, *n* = 12, *t*_(11)_ = 2.672, *P* = 0.0217. Paired student’s *t*-test) in PL PV interneurons, but PPR ratio (**k**, *n* = 12, *t*_(11)_ = 0.7976, *P* = 0.4420. Paired student’s *t*-test) was not altered. **m**, Representative traces of oEPSC of PL glutamatergic neurons in response to optical stimulation of CLA glutamatergic afferents before and after U50,488H application. **n**, **o**, U50,488H application decreased oEPSCs amplitude (**n**, *n* = 13, *t*_(12)_ = 3.393, *P* = 0.0053. Paired student’s *t*-test) and increased PPR ratio (**o**, *n* = 13, *t*_(12)_ = 2.541, *P* = 0.0259. Paired student’s *t*-test) in PL glutamatergic neurons. All data are shown as mean ± s.e.m. ^*^*P* < 0.05, ^**^*P* < 0.01. Source data are provided as a Source Data file.


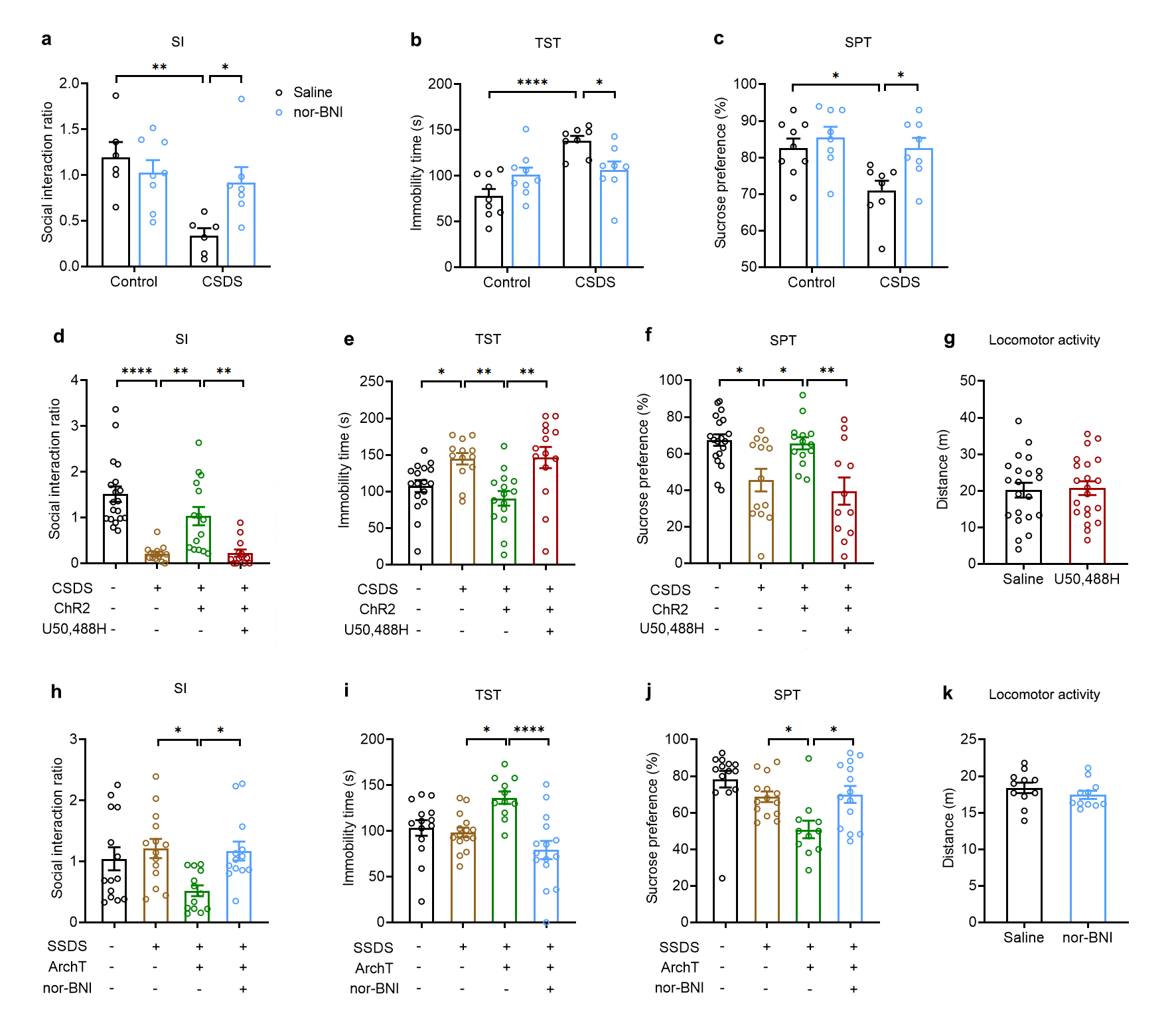


**Supplementary Fig. 11. KORs in CLA-PL circuit modulate depressive-like behaviors.**

**a-c**, Intra-PL infusion of nor-BNI increased the social interaction ratio (**a**, *n* = 6-8, *F*_(1,23)_ = 6.731, *P* = 0.0162. Two-way ANOVA), decreased the immobility time in the TST (**b**, *n* = 8-9, *F*_(1,30)_ = 12.37, *P* = 0.0014. Two-way ANOVA) and increased sucrose consumption in the SPT (**c**, *n* = 8-9, *F*_(1,29)_ = 2.498, *P* = 0.1249. Two-way ANOVA). **d-g**, Intra-PL infusion of KOR agonist U50,488H blocked photoactivation of CLA^Glu^-PL induced antidepressive-like effects. U50,488H blocked photo-activation of the CLA-PL pathway induced increase of social interaction (**d**, *n* = 13-19, *F*_(3,57)_ = 19.12, *P* < 0.0001. One-way ANOVA), decrease of immobility time in the TST (**e**, *n* = 13-17, *F*_(3,55)_ = 7.127, *P* = 0.0004. One-way ANOVA) and increase of sucrose consumption in the SPT (**f**, *n* = 12-19, *F*_(3,54)_ =8.166, *P* = 0.0001. One-way ANOVA) without affecting the locomotor activity (**g**, *n* = 21, *t*_(40)_ = 0.2068, *P* = 0.8372. Student’s *t*-test). **h-k**, Intra-PL infusion of KOR antagonist nor-BNI reversed photo-inhibition of CLA^Glu^-PL induced prodepressive-like behaviors. nor-BNI reversed photo-inhibition of the CLA-PL pathway induced decrease of social interaction ratio (**h**, *n* = 13-14, *F*_(3,50)_ = 4.205, *P* = 0.0099. One-way ANOVA), increase of immobility time in the TST (**i**, *n* = 11-15, *F*_(3,51)_ = 7.821, *P* = 0.0002. One-way ANOVA) and decrease of sucrose consumption in the SPT (**j**, *n* = 11-14, *F*_(3,49)_ = 6.731, *P* = 0.0007. One-way ANOVA) without affecting the locomotor activity (**k**, *n* = 11, *t*_(20)_ = 1.061, *P* = 0.3013. Student’s *t*-test). All data are shown as mean ± s.e.m. ^*^*P* < 0.05, ^**^*P* < 0.01, ^****^*P* < 0.0001. Source data are provided as a Source Data file.
